# Cardiomyopathy and Response to Enzyme Replacement Therapy in a Male Mouse Model for Fabry Disease

**DOI:** 10.1371/journal.pone.0033743

**Published:** 2012-05-04

**Authors:** Aurelie Nguyen Dinh Cat, Brigitte Escoubet, Vincent Agrapart, Violaine Griol-Charhbili, Trenton Schoeb, Wenguang Feng, Edgar Jaimes, David G. Warnock, Frederic Jaisser

**Affiliations:** 1 Inserm U872 Team 1, Paris, France; 2 University Pierre et Marie Curie, Paris, France; 3 AP-HP Hôpital Bichat, Paris, France; 4 Centre d’Explorations Fonctionnelles-Imagerie, Bichat Federative Research Institute 02, University Denis Diderot, Paris, France; 5 University of Alabama at Birmingham, Birmingham, Alabama, United States of America; Baylor Research Institute, United States of America

## Abstract

Fabry disease is an X-linked disorder of glycosphingolipid metabolism that results in progressive accumulation of neutral glycosphingolipids, (predominately globotriaosylceramide; GL-3) in lysosomes, as well as other cellular compartments and the extracellular space. Our aim was to characterize the cardiac phenotype of male knock-out mice that are deficient in alpha-galactosidase A activity, as a model for Fabry disease and test the efficacy of Enzyme Replacement Therapy with agalsidase-beta. Male mice (3–4 months of age) were characterized with awake blood pressure and heart rate measurements, cardiac echocardiography and electrocardiography measurements under light anesthesia, histological studies and molecular studies with real-time polymerase chain reaction. The Fabry knock-out mouse has bradycardia and lower blood pressure than control wild type (CB7BL/6J) mice. In Fabry knock-out mice, the cardiomyopathy associated mild hypertrophy at echography with normal systolic LV function and mild diastolic dysfunction. Premature atrial contractions were more frequent in without conduction defect. Heart weight normalized to tibial length was increased in Fabry knock-out mice. Ascending aorta dilatation was observed. Molecular studies were consistent with early stages of cardiac remodeling. A single dose of agalsidase-beta (3 mg/kg) did not affect the LV hypertrophy, function or heart rate, but did improve the mRNA signals of early cardiac remodeling. In conclusion, the alpha-galactosidase A deficient mice at 3 to 4 months of age have cardiac and vascular alterations similar to that described in early clinical stage of Fabry disease in children and adolescents. Enzyme replacement therapy affects cardiac molecular remodeling after a single dose.

## Introduction

After initial clinical descriptions, mutations in the alpha-galactosidase A (AGAL) gene were found to be responsible for Fabry disease, which is an X-linked disorder of glycosphingolipid metabolism that results in progressive accumulation of neutral glycosphingolipids, (predominately globotriaosylceramide; GL-3) in lysosomes, as well as other cellular compartments and the extracellular space [1]. The prevalence of Fabry mutation ranges from 1 in 40,000 to 1∶117,000 in United States and Australia to 1∶833,000 in Northern Portugal, the majority of them Caucasians [2]. These figures may underestimate the real prevalence of the disease as many patients go undiagnosed due to rarity of this disorder and phenotypic variation of the clinical features, especially in females. Much higher estimates of prevalence (e.g., 1 in 4,000) have been obtained from a newborn screening project, most of which were so-called “late-onset” variants with some residual enzyme activity [3]. Most affected males have little, if any, alpha-galactosidase A activity, and the deposition of GL-3 occurs primarily in vascular endothelial cells as well as epithelial and smooth muscle cells throughout the body. Early clinical manifestations of the disease include angiokeratoma, acroparesthesias, episodic pain “crises”, hypohydrosis, and gastrointestinal complaints [1].

Progressive GL-3 accumulation in the microvasculature and parenchyma leads to microvascular dysfunction, occlusion, and ischemia. Recent reports have described increased inflammation, oxidative stress, and circulating myeloperoxidase [4] which appears to be associated with vasculopathic events [5]. In adult males with Fabry disease, the renal, cardiovascular and cerebrovascular manifestations such as proteinuria, chronic kidney disease and kidney failure, cardiac arrhythmias, hypertrophic cardiomyopathy and strokes lead to early death during the fourth and fifth decade of life [1], [6], [7]. A late onset cardiac variant has been described in male patients which is associated with progressive cardiac fibrosis and ultimate death in the 6^th^ decade of life from the cardiac disease with preserved renal function [8], [9].

Recent studies have emphasized the importance of controlling proteinuria with inhibitors of the renin-angiotensin-aldosterone system in patients receiving enzyme replacement therapy (ERT) [10] but even with stabilization of kidney function, some of these patients still experience cardiac events, including bradyarrhythmias, ventricular premature contractions and sustained ventricular arrhythmias and conduction delays [11], [12] as have been described in untreated patients [8], [13]. The cardiac manifestations in adults with Fabry disease, with emphasis on the non-obstructive, concentric hypertrophic cardiomyopathy are well described [8], [14], [15], [16]. Kampmann *et al.* have studied a large number of adolescents with Fabry disease; some present with early symptoms and signs of cardiac involvement [17], findings that have been confirmed by reports from Fabry registries [18], [19].

Mouse knock-out (KO) models for Fabry disease have been described [20], [21]. Shayman *et al.* have studied large vessel reactivity and pathology in this model [4], [22], [23], [24], [25]. Recent work by Rozenfeld et al has described myocardial alterations in this model, and the response to ERT given at biweekly intervals for 2 months [26]. In the present study, we found that Fabry KO male mice have bradycardia, low systemic blood pressure and mild hypertrophic cardiomyopathy when compared to the control wild-type (WT) C57BL/6J mice. Molecular studies are consistent with early cardiac remodelling, and these changes were reversed rapidly in response to a single dose of ERT.

## Methods

### Ethics Statement

This study conforms to European Union Council Directives (86/609/EEC) regarding the care and use of laboratory animals, and the Guide for the Care and Use of Laboratory Animals published by the US National Institutes of Health (NIH Publication No. 85–23, revised 1996. The Institutional Animal Care and Use Committee approved live animal procedures conducted at the University of Alabama at Birmingham (Animal Project Number 10XX08339).

### Animal Model

Breeding pairs of the Fabry KO mouse were obtained from the National Institutes of Health (Bethesda, MD). This model as been previously used by Eitzman et al. to analyze vascular function [22]. Control WT animals were gender- and age-matched C57BL/6J mice obtained from the Charles River Laboratories. Male animals were used, and mice were provided standard chow (A04, Scientific Animal Food Engineering, Epinay sur Orge, France) and drank tap water *ad libitum*.

### Blood Pressure, Electrocardiography and Echocardiography Measurements

Systolic blood pressure (SBP) on trained conscious mice was measured by tail cuff plethysmography using a BP2000 Visitech model as published previously [27]. Conscious heart rate (HR) was extracted from the pulse signal. Electrocardiograms (ECGs) were recorded over a 10 min period in 3–4-month-old mice under light anesthesia with isoflurane. Arrhythmia was detected by analysis of the tachogram of the recording (10 min tracings). Tachograms were constructed from automatic R wave detection (ECG-Auto EMKA, France) and RR plotted against time. All abnormal RR intervals were checked for validation and labeling. ECG intervals were measured from short recordings on average beats, constructed from 200 consecutive QRST complexes. Intervals were determined semi-automatically from a library of QRST waveforms sampled from the tracing. Average beats were checked and intervals set using the first derivative of the tracing. PQ was measured from the onset of P wave to the onset of the QRS wave. QRS duration was measured from the onset of the Q wave to the end of high-amplitude electrical events, as detected on the first derivative. The QT interval was measured from the onset of the Q wave to the last detectable electrical event on the first derivative. QT interval was corrected for heart rate by drawing the linear regression line from individual beats for each mouse, and was expressed as the value at a RR of 150 msec [28].

Echocardiography was performed on lightly anesthetized mice (isoflurane, Abbott, in oxygen), as described previously [29]. A small number of animals appeared to have more severe bradycardia in response anesthesia, and these animals were not included in the analysis of the echocardiograhy results to avoid non-specific rate-related changes. Briefly, the heart was visualized in the long axis parasternal view for M-mode left ventricle (LV) dimension measurement and posterior wall pulse wave tissue Doppler measurement. An apical 4- to 5-chamber view was obtained from the subcostal view for diastolic function assessment with pulse wave spectral LV inflow and outflow and for pulse wave tissue Doppler measurement of the mitral annulus velocities [29]. Relative wall thickness was calculated as (IVST+LVPWT)/LV EDD (IVST, interventricular wall thickness; LVPWT, LV posterior wall thickness).

### Histochemistry and Quantification of Non-Vascular Collagen

Following intra-aortic perfusion with phosphate-buffered saline, hearts were excised from mice anesthetized with pentobarbital (50 mg/kg, i.p.) and fixed in 10% neutral buffered formalin. Fixed hearts were transected perpendicular to the long axis through the ventricles at their widest point and processed routinely for paraffin sectioning. Both portions were embedded so that sections contained 2 cross sections. Sections were cut 5 µm thick and stained with hematoxylin and eosin or Pirosirius Red [30]. Fields of the anterior, posterior and lateral left ventricle and the intraventricular septum were digitally photographed at 10x objective magnification with routine transmitted light and with polarization. For each field, myocardial area was determined by extraction of the green color channel, thresholding, and measurement with Image Pro Plus 6.2 software (MediaCybernetics, Inc. Bethesda MD). Myocardial collagen was determined by inverting polarized images, thresholding, deleting normal perivascular collagenous tissue and measurement of remaining areas. Collagen area was expressed as a percentage of the total myocardial area for each field. Myocyte diameters were measured perpendicular to the long axis of the sarcomeres from unbranched areas of the myocytes near an intercalated disk.

### RNA Purification, cDNA Synthesis and Reverse Transcriptase-Polymerase Chain Reaction (RT-PCR)

Total RNA was extracted from the hearts of control and Fabry KO mice after being homogenized in Trizol (Trisol Reagent, Invitrogen), following the instructions of the manufacturer. Total RNA samples were treated with DNase I (Ambion®, Applied Biosystems, Courtaboeuf, France) and reverse-transcribed using random hexamers (Invitrogen, Cergy Pontoise, France) and the DNA polymerase SuperScriptII (Invitrogen). Real-time PCR was carried out on the iCycler (Biorad) using gene-specific primers to quantify the relative abundance of each gene with SYBR Green I as the fluorescent molecule as described [31]. The primers used are listed in Table S1. The GAPDH gene was used as reference gene for normalization. Relative copy number of the target genes was calculated with the 2^(–ΔΔCt)^ method, as described [31].

### Enzyme Replacement Therapy (ERT)

Mice were inject via tail vein with a single injection of agalsidase-beta (Fabrazyme®, Genzyme Corporation, Cambridge MA, USA) at a dose of 3 mg/kg, as previously described [20]; GL-3 levels in the heart reached a nadir at 3 weeks following a single intravenous injection of agalsidase-beta at 3 mg/kg, which is the timing and dose of agalsidase-beta used in the present study. Control animals received similar volumes of normal saline. Mice underwent physiologic and echocardiographic assessment prior to injection, and then at 3 weeks following injection, at which point the cardiac GL-3 content was stably reduced by 80% [20].

### Statistics

Data are expressed as mean ± SE. Student’s two-tailed *t* tests were used to compare unpaired data between two groups. If the global test was significant, pair-wise comparisons were performed with a Tuckey-Kramer test. *P* * 0.05 was considered significant.

## Results

### Systolic Blood Pressure, Heart Rate, ECG and Cardiac Weight Measurements

**Figure 1:**
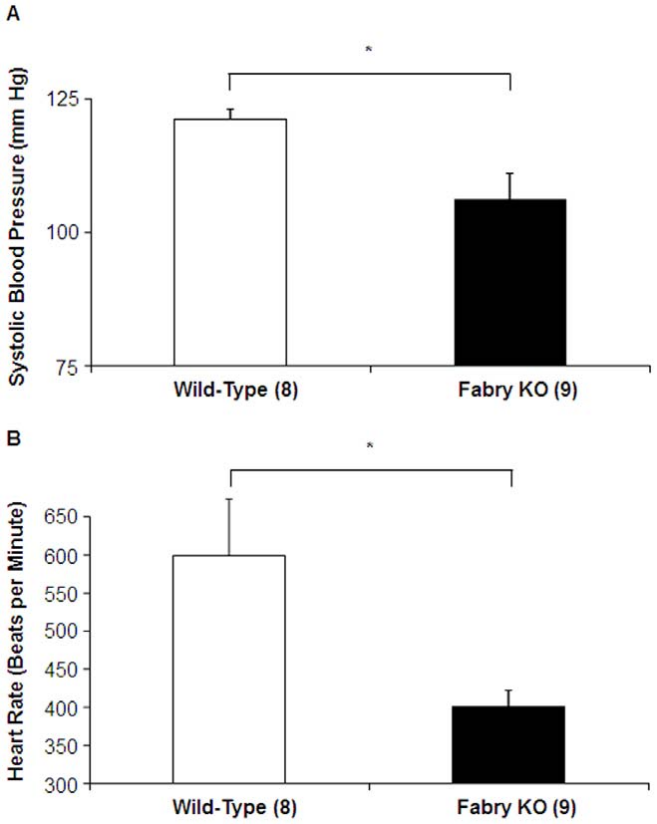
Comparison of Fabry Knock-out (KO) mice to wild type C57BL/6NJ mice: Tail Cuff Measurements. (A) Systolic blood pressures (mm Hg) and (B) Heart rates (beats per minute) were determined with tail-cuff measurements in awake animals. Data represent the means ± SE for 9 Fabry KO and 8 wild type male mice. Statistical significance was determined by unpaired, two-tailed *t*-test: *P<0.05.

Systolic blood pressure was lower for male Fabry KO mice than for male wild-type mice (WT) (Figure *1A*). In addition, heart rate was significantly slower in the Fabry KO mice than the WT controls (*Figure 1B*).

The measurements of RR intervals with surface ECG recordings showed prolonged RR intervals for Fabry KO mice compared to WT controls (Table 1). and the standard deviations of the RR intervals (SDNN) were significantly increased in the Fabry KO mice compared to the WT controls after normalization for heart rate (Fabry KO: 12% vs WT: 5%). There were no differences in PQ, QRS, or corrected QT intervals (Table 1). Premature atrial contractions were more frequently observed in Fabry KO mice than WT mice (Table 1).

**Table 1:** ECG results in 3–4 month old WT and Fabry KO mice.

	WT Controls	Fabry KO
Number	12	16
RR interval (msec)	117±4.5	135±5.6*
SDNN (msec)	6.4±0.7	16.2±1.4*
PR interval (msec)	36±0.4	37±0.8
QRS interval (msec)	16±0.5	16±0. 6
QT_c150_ interval (msec)	49±1.2	47±2.0
APC (%)	33	93**

Surface ECGs were obtained in lightly anesthesized mice. Data represent the means ± SE. Statistical significance was determined by pair-wise comparisons with a Tuckey-Kramer test, or χ^2^ analysis.

WT, wild-type; KO, knockout; SDNN, standard deviation of normal RR intervals; APC, %of animals with atrial premature contractions during 10 min recording.

* P<0.05,

** P<0.05 by for χ^2^ analysis.

**Figure 2:**
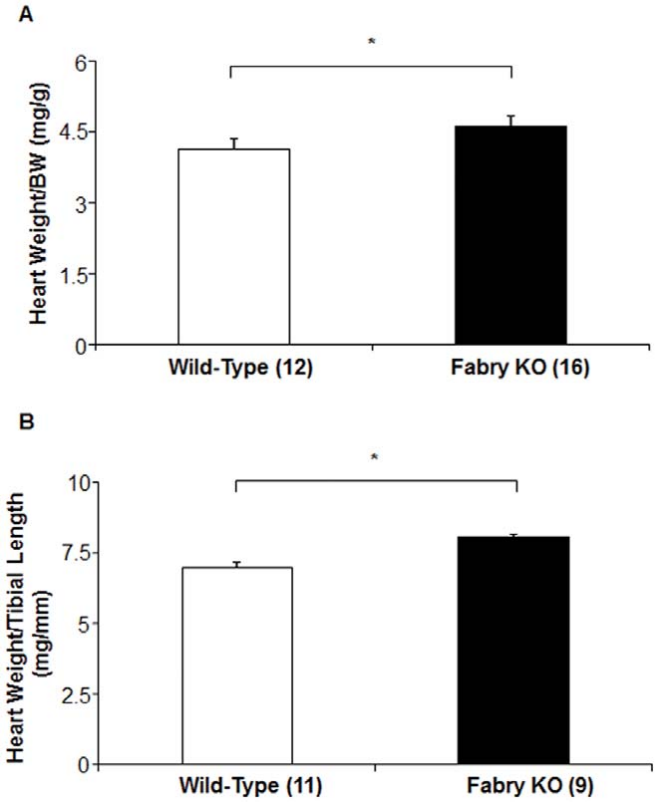
Comparison of Fabry Knock-out (KO) mice to wild type C57BL/6NJ mice: Heart Weights. Body weights (g), heart weights (mg) and tibial lengths (mm) were measured at sacrifice. Data represent the means ± SE for 9 Fabry KO and 11 wild type male mice. Statistical significance was determined by unpaired, two-tailed *t*-test: *P<0.05.

Heart weight was increased for Fabry KO mice, compared to WT mice when normalized to body weight (Figure *2A*) or tibial length (*Figure 2B*).

### Left Ventricle and Aortic Structural Alterations

The echocardiography results for 4 month-old mice are summarized in Table 2. There were significant increases in LV mass normalized to body weight (LV mass/BW) for Fabry KO mice compared to the WT age-matched controls (4.8±0.32 versus 4.2±0.14 mg/g; p<0.05). Despite the lower systolic blood pressure measurements, there was also a significant increase in the aortic diameter during diastole for Fabry KO mice compared to the WT controls (1.6±0.07 versus 1.4±0.03 mm; p<0.01). Aortic dilation was not associated with aortic valvular regurgitation (data not shown).

**Table 2:** Echocardiographic evaluation of 3 to 4 month-old Wild type and Fabry Knock Out (KO) mice.

	Wild Type	Fabry KO
Number	12	11
Body Weight (g)	27.6±0.4	31.1±0.5
Age (weeks)	18.8±0.1	18.2±0.3
Heart Rate (beats per min)	518±7	521±9.4
*Cardiac and vascular remodeling*		
LA Diameter (mm)	2.2±0.08	2.7±0.10
LA Diameter/BW (mm/g)	0.1±0.01	0.1±0.03
LV EDD/BW (mm/g)	0.15±0.01	0.15±0.01
LV mass/BW (mg/g)	4.2±0.14	4.8±0.32*
Relative Wall Thickness	0.4±0.02	0.4±0.02
Diastolic Ao (mm)	1.4±0.03	1.6±0.07**
*Left Ventricular Systolic Function*		
LV Ejection Fraction (%)	82±2	79±3
Vcfc, circumference/sec	3.25±0.12	3.15±0.31
Sa (cm/s)	2.7±0.20	2.9±0.12
Spw (cm/s)	3.1±0.20	3.2±0.16
*Left Ventricular Diastolic Function*		
IVRT (ms)	17.0±0.60	17.0±1.1
Ea (cm/s)	5.5±0.24	4.5±0.40**
Epw (cm/s)	4.4±0.19	4.0±0.40
E/Ea	19.1±0.8	21.7±1.7

Data represent the means ± SE. Statistical significance was determined by unpaired, two-tailed *t*-test: *P<0.05: **P<0.01.

Abbreviations: LA, left atrium; LV, left ventricle; LV EDD, left ventricular end diastolic diameter; Vcfc, velocity of shortening of circumferential fibers; Sa, Spw: maximal systolic velocity of the mitral annulus and posterior wall; IVRT: isovolumic relaxation time; Ea and Epw: maximal diastolic velocity of the mitral annulus and posterior wall; E, maximal velocity of LV inflow.

### Left Ventricular Functional Alteration

Global LV systolic function was similar in Fabry KO mice as compared to age-matched WT mice, as assessed with echographic analysis of ejection fraction and systolic velocity of the mitral annulus by tissue Doppler (Table 2). Fabry KO mice had mild diastolic LV dysfunction as depicted with the decrease in Ea velocity, without change in the isovolumic relaxation time.

### Left Ventricular Histologic Alteration

**Figure 3:**
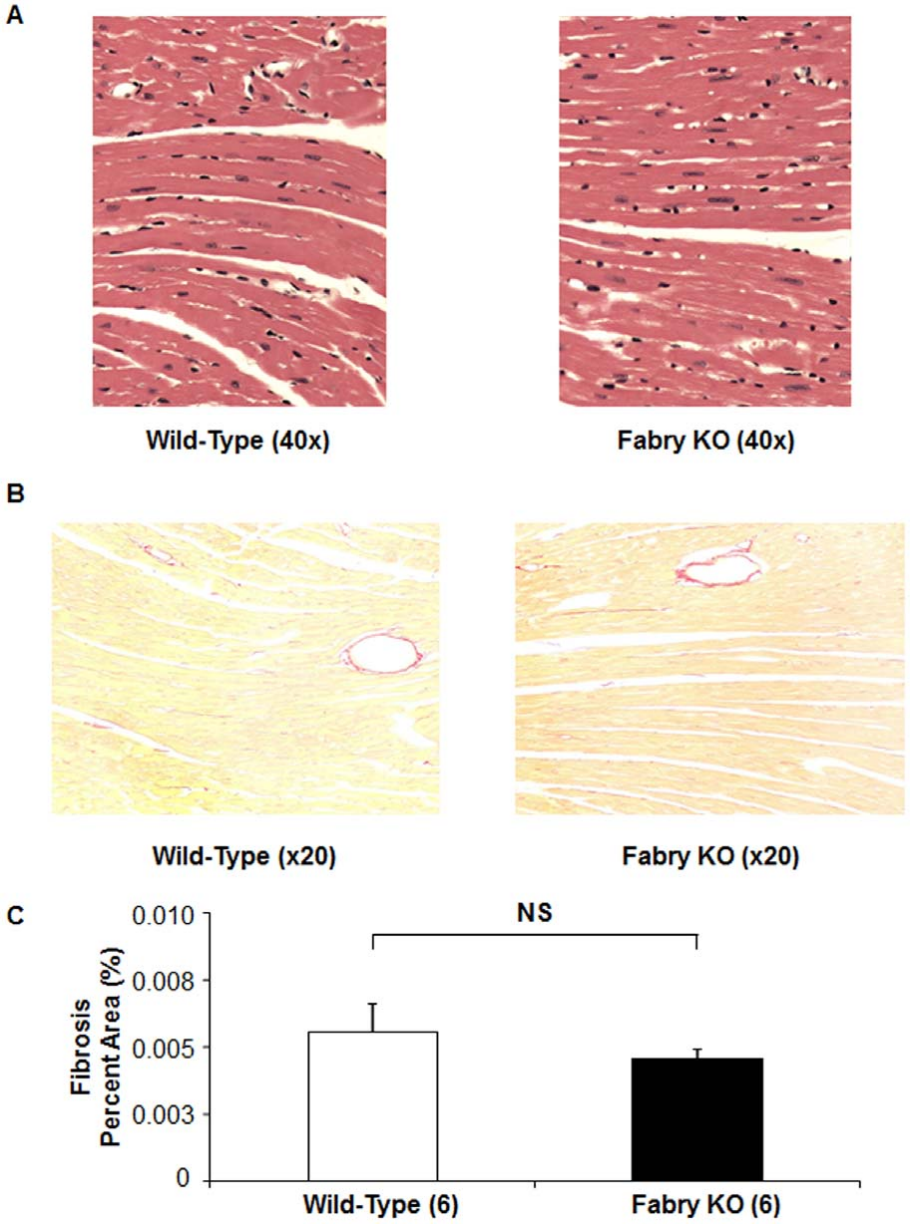
Left Ventricular Histology of wild type C57BL/6NJ mice to Fabry Knock-out (KO) mice. (A) Wild type mouse, left panel; Fabry KO mouse, right panel; 40×magnification. Hearts were perfusion fixed and sections obtained for staining for hematoxylin and eosin. (B) Pirosirius Red staining for non-vascular collagen (Wild type mouse, left panel; Fabry KO mouse, right panel; 20×magnification). (C) Pirosirius Red staining for non-vascular collagen, expressed as the percentage of the total myocardial area for each field.

Routine histological stains (*Figure 3A*) did not reveal any structural difference between 4 month-old Fabry KO and wild type mice. Morphometric analysis showed identical myocyte diameters (Table 3), and electron micrographs showed occasional electron dense lamelated inclusion bodies, similar to previous descriptions (data not shown) [20].

Staining with Picrosirius Red showed no significant differences in non-vascular collagen staining in the myocardium of male Fabry KO mice compared to WT mice (*Figure 3C–B*) and no predominant area of fibrosis in Fabry KO mice.

**Table 3:** Myocyte Diameter and Picrosirius Red Staining of 4-month old Fabry KO and WT mice.

	WT Controls	Fabry KOs
Myocyte diameter (µm)	82.6±9.9	79.1±4.9
Collagen content (%)	0.0056±0.0025	0.0046±0.0008

### LV Molecular Alterations in Fabry KO Mice

**Figure 4:**
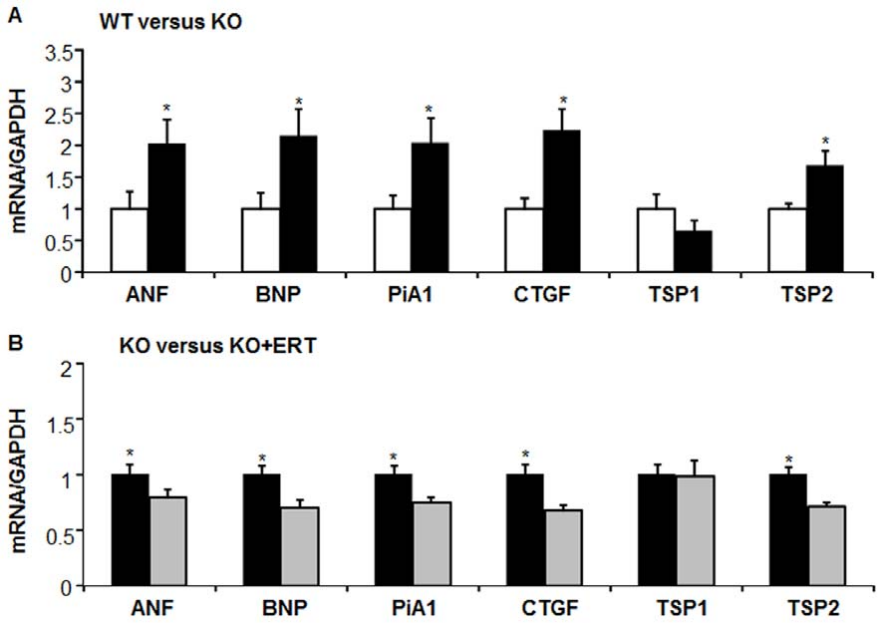
Comparison of wild type C57BL/6NJ mice (open bars) to Fabry Knock-out (KO) mice (black bars): mRNA levels. Real-time PCR amplification of indicated mRNA targets, normalized to GAPDH mRNA level. ANF, atrial natriuretic factor; BNP, brain natriuretic peptide; PAI-1, plasminogen activator inhibitor -1; CTGF, connective tissue growth factor; TSP1, thrombospondin 1; TSP2, thrombospondin 2; Statistical significance was determined by unpaired, two-tailed *t*-test: *P<0.05. (A). Comparison of wild type C57BL/6NJ mice (open bars) to Fabry Knock-out (KO) mice (black bars): mRNA levels. (B). Comparison of Fabry Knock-out (KO) mice (black bars) to Fabry Knock-out (KO) mice (gray bars) 3 weeks after a single intravenous treatment with agalsidase-beta at 3 mg/kg: mRNA levels.

We examined myocardial alterations that could be associated with cardiac remodeling and dysfunction by analyzing mRNA expression for atrial natriuretic factor (ANF) brain natriuretic peptide (BNP), plasminogen activator inhibitor-1 (PAI-1); connective tissue growth factor (CTGF), thrombospondin 1 (TSP1), and thrombospondin 2 (TSP2). As shown in Figure 4A, ANF and BNP mRNA levels normalized to GAPDH levels were significantly increased in male Fabry KO mice compared to wild type controls. PAI-1 and CTGF mRNA levels were increased in male Fabry KO mice, as compared to wild type. Also shown in Figure 4 are the normalized values for TSP1 and TSP2, members of the matricellular protein family, which are up-regulated during cardiac stress, injury and remodeling [32]. TSP2 but not TSP1 mRNA levels were increased in male Fabry KO mice compared to wild type controls. The mRNA levels for collagen 1a and collagen 3a, the matrix metallo-proteinases 2 and 9 were not altered in Fabry KO mice (data not shown).

### Systolic Blood Pressure, Heart Rate, ECG and Cardiac Weight Measurements: Effects of ERT

The measurements of RR intervals with surface ECG recordings showed identical RR intervals and the standard deviation of the RR intervals, and the frequency of premature atrial contractions for Fabry KO mice compared to Fabry KO mice treated with 3 mg/kg intravenous ERT 3 weeks before (Table 4). There were no significant differences in PQ, QRS, or corrected QT intervals (data not shown).

**Table 4:** ECG results in 6–7 month old Fabry KO mice: effects of ERT.

	KO Controls	KO ± ERT
Number	11	11
RR (msec)	135±5.6	143±4
SDNN (ms)	16.7±2	19.7±2.2
APC (%)	45.6	54

Surface ECGs were obtained in lightly anesthesized mice. Data represent the means ± SE. Statistical significance was determined by pair-wise comparisons with a Tuckey-Kramer test, or χ^2^ analysis.

WT, wild-type; KO, knockout; SDNN, standard deviation of normal RR intervals;APC, % of animals with atrial premature contractions during 10 min recording.

**Figure 5:**
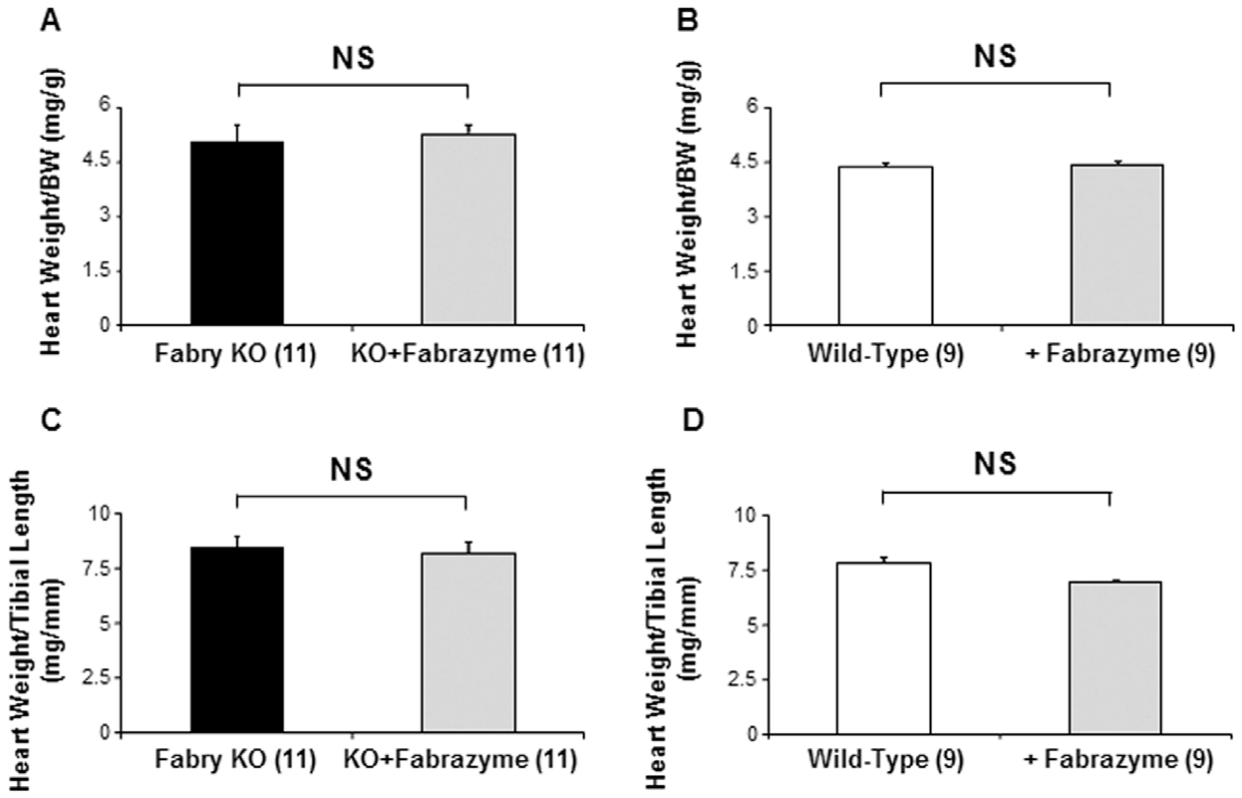
Comparison of untreated Fabry Knock-out (KO) and WT mice to ERT-treated KO mice and ERT-treated WT mice: Heart Weights. Body weights (g), heart weights (mg) and tibial lengths (mm) were measured at sacrifice. Data represent the means ± SE for 11 Fabry KO and 9 wild type male mice. Statistical significance was determined by unpaired, two-tailed *t*-test: *P<0.05.

ERT had no effect 3 weeks after injection on heart weight for Fabry KO mice, when normalized to body weight (Figure *5A*) or tibial length (*Figure 5*C). There was no effect 3 weeks after injection on heart weight for WT mice, when normalized to body weight (Figure *5B*) or tibial length (*Figure 5D*).

### Left Ventricle and Aortic Structural Alterations with ERT

The echocardiography results for 6–7 month-old Fabry KO mice are summarized in Table 5 for untreated mice and those who received a single injection of ERT 3 weeks before evaluation. While there was no significant effect on the LV mass estimates, there was a significant increase in relative wall thickness for Fabry KO mice treated with ERT compared to the untreated KO age-matched controls according to a trend toward decrease in LV diameter after treatment (3.9±0.6 vs 4.2±0.15, ns) without change in wall thickness (septum: Fabry mice ERT 1.0±0.03, Fabry mice: 0.9±0.02; posterior wall Fabry mice ERT 0.8±0.04, Fabry mice: 0.7±0.03). There was a significant decrease in the aortic diameter during diastole for Fabry KO mice treated with ERT compared to the age-matched KO controls (1.7±0.05 versus 1.6±0.04 mm; p<0.01).

**Table 5:** Echocardiographic evaluation of 6–7 month-old Fabry Knock Out (KO) mice: response to enzyme replacement therapy (ERT).

	Fabry KO	Fabry KO + ERT
Number	11	11
Body Weight (g)	31.1±0.5	29.6±0.6
Age (weeks)	28	27
Heart Rate (beats per min)	521±9.4	530±12
*Cardiac and vascular remodeling*		
LA Diameter (mm)	2.7±0.19	2.4±0.13
LA Diameter/BW (mm/g)	0.1±0.03	0.1±0.03
LV EDD/BW (mm/g)	0.1±0.01	0.1±0.01
LV mass/BW (mg/g)	4.4±0.22	4.6±0.16
Relative Wall Thickness	0.4±0.02	0.5±0.02*
Diastolic Ao (mm)	1.7±0.05	1.6±0.04*
*Left Ventricular Systolic Function*		
LV Ejection Fraction (%)	84±2	81±2
Vcfc, circumference/sec	3.41±0.20	3.33±0.18
Sa (cm/s)	2.90±0.12	3.13±0.10
Spw (cm/s)	3.4±0.23	3.3±0.17
*Left Ventricular Diastolic Function*		
IVRT (ms)	17.0±0.54	17.8±0.83
Ea (cm/s)	5.0±0.15	5.0±0.19
Epw (cm/s)	4.1±0.18	4.8±0.27
E/Ea	19.5±0.78	19.0±0.71

Data represent the means ± SE. Statistical significance was determined by unpaired, two-tailed *t*-test: *P<0.05.

Abbreviations: LA, left atrium; LV, left ventricle; LV EDD, left ventricular end diastolic diameter; Vcfc, velocity of shortening of circumferential fibers; Sa, Spw: maximal systolic velocity of the mitral annulus and posterior wall; IVRT: isovolumic relaxation time; Ea and Epw: maximal diastolic velocity of the mitral annulus and posterior wall; E, maximal velocity of LV inflow.

### Left Ventricular Functional Alterations in Fabry KO Mice with ERT

The heart rates obtained in 6–7 week old mice undergoing echocardiography with isoflurane anesthesia were the same for untreated Fabry KO mice compared to age-matched Fabry KO mice that had received ERT (3 mg/kg) as a single IV injection 3 weeks before evaluation (Table 5).

LV systolic function, as assessed by echocardiography was similar in ERT-treated Fabry KO mice as compared to age-matched untreated KO mice (Table 4). ERT-treated Fabry KO mice had similar diastolic LV function as the untreated KO controls.

### LV Molecular Alterations in Fabry KO and WT Mice Treated with ERT

As shown in Figure 4B, ANF and BNP mRNA levels normalized to GAPDH levels were significantly decreased in male Fabry KO mice 3 weeks after ERT treatment compared to untreated KO controls. PAI-1 and CTGF mRNA levels were decreased in ERT-treated male Fabry KO mice, as compared to untreated KO controls. TSP2 but not TSP1 mRNA levels were decreased in ERT-treated male Fabry KO mice compared to untreated KO controls.

The effects of ERT were also examined in WT controls 3 weeks after a single IV injection (3 mg/kg), compared to untreated age-matched WT controls. There was a significant decrease in BNP mRNA normalized to GAPDH levels in ERT-treated WT mice, but there were not any effects of ERT on any of the mRNA levels examined in this series compared to WT concurrent control male mice (Figure S1).

## Discussion

In the present study, we define the cardiac phenotype in male Fabry KO mice. We found that these mice have bradycardia and low systemic blood pressures compared to the control WT strain, as well as mild hypertrophic cardiomyopathy on echocardiography and gravimetry. The heart rates and systolic blood pressures reported herein for the WT mice are very similar to previous reports [33], [34].

Thus KO Fabry mice displayed a mild hypertrophic cardiomyopathy with structural and functional alteration similar to that described for the early stages of human myocardiopathies. Our results are similar to and extend the findings of Rozenfeld et al., who have described changes in passive pressure volume curves and contractility measurements in the male mouse KO model of Fabry disease, but did not document hypertrophy as assessed by cardiac weight normalized to tibial length or echo cardiographic assessment of left ventricular mass [26].

The initial descriptions of the KO model of Fabry disease were used for pharmacokinetic studies of ERT, and did not evaluate the phenotype of these animals (“.no clinical disease phenotype and survived a normal laboratory lifespan”[20]). Shayman et al. have studied large vessel reactivity and pathology in this model [4], [22], [23], [24], [25], [35] but did not evaluated the cardiac findings.

The most striking aspect of the cardiac phenotype in the KO mice was hypotension and bradycardia, compared to the WT controls. Autonomic nervous system alteration, as assessed on heart rate variability, was described in children and adolescents with decreased heart rate variability in boys [17] and could represent early findings of the cardiac involvement in Fabry disease. Our results suggest that the mouse model have also alteration of the autonomic nervous system, as depicted by bradycardia and time domain parameters of heart rate variability suggesting increase in variability. The change in time domain parameter (SDNN) could be partly accounted for by bradycardia itself with increasing dispersion of the RR but also by a greater influence of parasympathetic activity which could also be responsible for the lower blood pressure. Comparison with the human findings would need a complete autononic nervous system evaluation in mice.

Endothelial dysfunction was described in human Fabry disease with inflammation and increased oxidative stress within the vessel wall [4]. GL-3 accumulation occurs primarily in endothelial cells with impairment of vessel reactivity [4], [25]. Alteration in endothelial function, with the consequent change in smooth muscle cells, could be related to our findings of ascending aorta dilatation in the KO model that does not appear to be explained by hemodynamic changes. This has also been recently described in the human disease [36].

Cardiac abnormalities in Fabry disease are characterized mainly by LV wall thickening without significant dilatation, and LV hypertrophy [14]. Systolic function is largely preserved in most affected individuals, but diastolic dysfunction is a relatively common finding even at early stage of the disease [14], [17]. In the mouse model, we found LV functional alterations with mild alteration of diastolic function, as depicted with Tissue Doppler interrogation, and no systolic alteration. This is very similar to the findings in human at early stages of the cardiac disease [37], [38], [39]. Moreover, a good relationship was shown between tissue Doppler echocardiographic data and mechanical properties of human cardiac myofilaments obtained from myocardial biopsies [38]. The findings that the mouse model has mild cardiac involvement detectable with tissue Doppler suggest that this specific interrogation in human has to be performed for early detection of cardiac involvement, inasmuch as the efficiency of the replacement therapy is dependent on early onset of treatment [15]. We also found evidence of cardiac remodeling at the molecular level, with increased mRNA levels, for atrial natriuretic factor (ANF) brain natriuretic peptide (BNP), plasminogen activator inhibitor-1 (PAI-1); connective tissue growth factor (CTGF), and thrombospondin 2 (TSP2).

Our results suggest that atrial arrhythmias could be associated with early cardiac involvement in Fabry disease. Although more than 60% of patients with Fabry disease have evidence of cardiac involvement, the prevalence and clinical significance of arrhythmia in Fabry disease are unknown. Shah and colleagues studied 78 consecutive patients (mean age 43.5±15.0 years, 43 males) with Fabry disease [40]. Three patients (3.9%) had persistent atrial fibrillation, and 8 (13.3%) had paroxysmal atrial fibrillation. Five males (8.3%) had nonsustained ventricular tachycardia with a maximal left ventricular wall thickness >20 mm. Age, left atrial diameter, maximal left ventricular wall thickness, left ventricular mass index, and angina were univariate predictors of atrial fibrillation. During follow-up, there was 1 sudden cardiac death, 4 patients received pacemakers for bradyarrhythmias, and 1 patient received a biventricular pacemaker and an internal cardiac defibrillator. The high incidence of arrhythmias and pacemaker implantation and sudden cardiac death suggests that arrhythmia has a significant impact on the natural history of Fabry disease. The mouse model did not, however, displayed ventricular arrhythmias possibly because the mice were relatively young.

Despite the describe phenotype, the mouse model did not recapitulate all cardiac feature of human Fabry disease: 1) Ventricular arrhythmias or conduction (atrioventricular or intraventricular) defect were not found; 2) LV hypertrophy and GL-3 accumulation was mild or absent; and 3) Accelerated arteriosclerosis and vascular thrombosis was not evident in the mouse model, at least at the ages we examined in this series.

Over the last decade, the natural history of Fabry disease appears to have changed. With the use of ERT and antiproteinuric therapy, renal outcomes have improved and currently the most common cause of death is cardiac involvement and sudden cardiac death [41], [42] Electrocardiographic changes that have been described in Fabry disease include atrial fibrillation, atrioventricular conduction abnormalities, left ventricular hypertrophy, and repolarization abnormalities [17], [43]; [44]. Cardiac symptoms in patients with Fabry disease include bradycardia, shortness of breath with exertion, vasospastic and/or exertional angina pectoris, and syncope [45], [46]. Long term treatment with ERT has been shown to reduce LV hypertrophy, and suggested that early treatment may yield better long-term cardiac outcomes and exercise capacity [15].

Whether or not these abnormalities in heart rhythm or rate, as well as the cardiac remodeling will respond to ERT, and/or other forms of adjunctive therapy [47] are questions that can be addressed in the Fabry KO mouse despite the limitations of the model [1]. We have found changes in the cellular phenotype in this model, 3 weeks following a single intravenous injection of 3 mg/kg agalsidase-beta, a dose and interval chosen to maximize the reduction in cardiac GL3 content [20]. Rozenfeld et al. observed improvement in left ventricular contractility following longer treatment periods (4 injections over 2 months) of a much lower dose of ERT (0.5 mg/kg) [26]. Alternate dosing schedules as well as consideration of alternative forms of therapy [48] for this condition are important avenues for future research.

## Supporting Information

Figure S1 Comparison of WT mice treated with ERT to WT mice 3 weeks after a single intravenous injection with agalsidase-beta at 3 mg/kg: mRNA levels. Wild type C57BL/6NJ mice (open bars) compared to ERT-treated WT mice (gray bars). Real-time PCR amplification of indicated mRNA targets, normalized to GAPDH mRNA level. ANF, atrial natriuretic factor; BNP, brain natriuretic peptide; PAI-1, plasminogen activator inhibitor -1; CTGF, connective tissue growth factor; TSP1, thrombospondin 1; TSP2, thrombospondin 2; Statistical significance was determined by unpaired, two-tailed *t*-test: *P<0.05.
(TIF)

Table S1 Primers sequences.
(DOCX)
