# Supplementary figures and images for: Cardiomyopathy and Response to Enzyme Replacement Therapy in a Male Mouse Model for Fabry Disease
Source: PLoS One. 2012 May 4;7(5):e33743. doi: 10.1371/journal.pone.0033743 (PMC3344819; doi:10.1371/journal.pone.0033743)

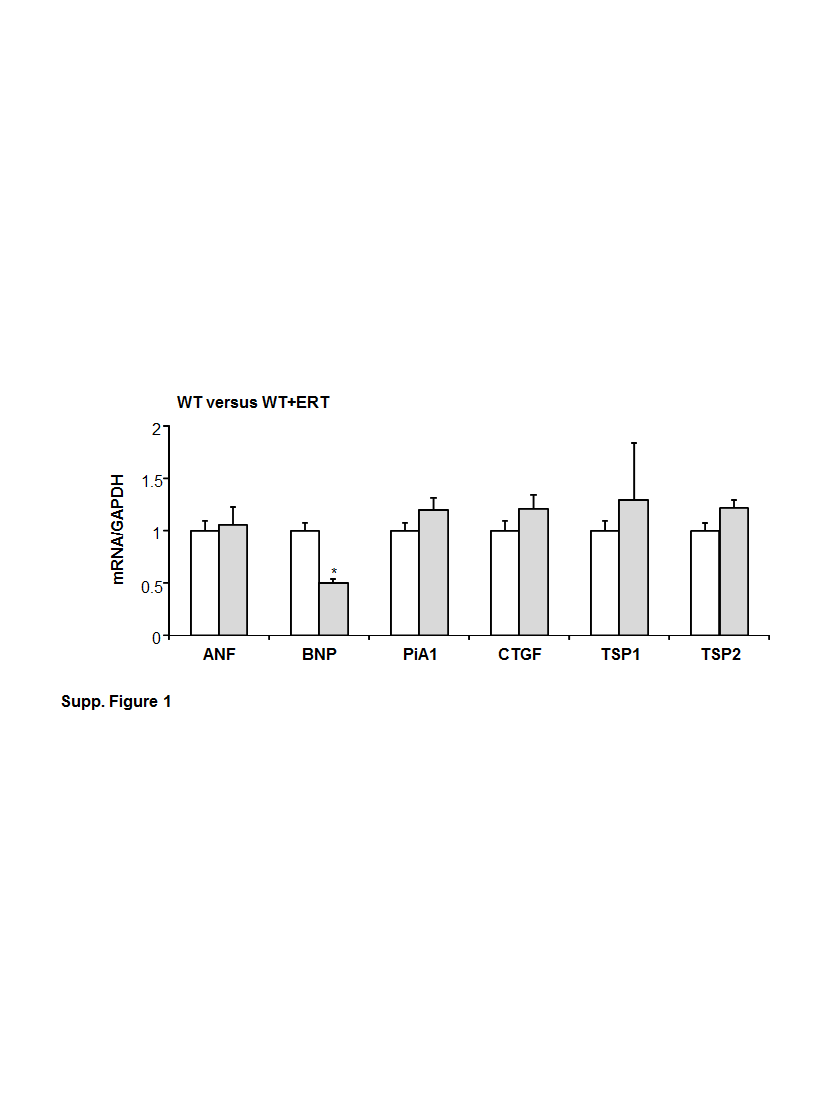

Supplement: Figure S1 — Comparison of WT mice treated with ERT to WT mice 3 weeks after a single intravenous injection with agalsidase-beta at 3 mg/kg: mRNA levels. Wild type C57BL/6NJ mice (open bars) compared to ERT-treated WT mice (gray bars). Real-time PCR amplification of indicated mRNA targets, normalized to GAPDH mRNA level. ANF, atrial natriuretic factor; BNP, brain natriuretic peptide; PAI-1, plasminogen activator inhibitor -1; CTGF, connective tissue growth factor; TSP1, thrombospondin 1; TSP2, thrombospondin 2; Statistical significance was determined by unpaired, two-tailed t-test: *P<0.05. (TIF) [file pone.0033743.s001.tif]
