# Supplementary material for: Cardiomyopathy and Response to Enzyme Replacement Therapy in a Male Mouse Model for Fabry Disease
Source: PLoS One. 2012 May 4;7(5):e33743. doi: 10.1371/journal.pone.0033743 (PMC3344819; doi:10.1371/journal.pone.0033743)
Supplement: Table S1 — Primers sequences. (DOCX) [file pone.0033743.s002.docx]

**Tables**

Table 1 Primers sequences

| **Genes** | **Forward Primer** | **Reverse Primer** |
| --- | --- | --- |
| GAPDH | AATGGTGAAGGTCGGTGTG | GAAGATGGTGATGGGCTTCC |
| ANP | CTCGTCTTGGCCTTTTGG | TCGGGGAGGAGGGAGCTAAGT |
| BNP | CGTCAGTCGTTTGGGCTGTA | GCAGCCAGGCGGTCTTCCT |
| PAI1 | AGGCACTGCAAAAGGTCAGGATCGA | GGCCATGCGGGCTGAGATGA |
| CTGF | TGACCCCTGCGACCCACA | TACACCGACCCACCGAAGACACAG |
| TSP-1 | GCGTTGCCAGGCTCCGAGTT | AGCTGAGCTGGAGCAGCCTT |
| TSP-2 | AGCGGCCGGGAAACCAAACC | AGCGCTCACGGATCCCTCCA |

GAPDH: glyceraldehyde-3-phosphate dehydrogenase, ANP: natriuretic peptide precursor type A, BNP: natriuretic peptide precursor type B ubiquitin, PAI1: serine peptidase inhibiteur, CTGF: connective tissue growth factor, TSP-1: Thrombospondin-2, TSP-1: Thrombospondin-
